# Tunable Magnetic Properties of Interconnected Permalloy Nanowire Networks

**DOI:** 10.3390/nano13131971

**Published:** 2023-06-29

**Authors:** Alejandro Pereira, Guidobeth Sáez, Eduardo Saavedra, Juan Escrig

**Affiliations:** 1 Department of Sciences, Faculty of Liberal Arts, Adolfo Ibañez University, Santiago 7941169, Chile; alejandro.pereira@uai.cl; 2 Department of Physics, Faculty of Physical and Mathematical Sciences, University of Chile, Santiago 8370448, Chile; guidobethsaez@ug.uchile.cl; 3 Department of Physics, University of Santiago de Chile (USACH), Santiago 9170124, Chile; eduardo.saavedra.d@usach.cl; 4 Center for the Development of Nanoscience and Nanotechnology (CEDENNA), Santiago 9170124, Chile

**Keywords:** magnetic properties, permalloy, interconnected nanowire networks, hysteresis curves

## Abstract

In this study, we investigate the magnetic properties of interconnected permalloy nanowire networks using micromagnetic simulations. The effects of interconnectivity on the hysteresis curves, coercivity, and remanence of the nanowire networks are analyzed. Our results reveal intriguing characteristics of the hysteresis curves, including nonmonotonic behaviors of coercivity as a function of the position of horizontal nanowires relative to vertical nanowires. By introducing horizontal nanowires at specific positions, the coercivity of the nanowire networks can be enhanced without altering the material composition. The normalized remanence remains relatively constant regardless of the position of the horizontal wires, although it is lower in the interconnected nanowire arrays compared to nonconnected arrays. These findings provide valuable insights into the design and optimization of nanowire networks for applications requiring tailored magnetic properties.

## 1. Introduction

Permalloy, a ferromagnetic alloy composed primarily of iron and nickel, has garnered significant attention in the field of materials science and engineering due to its remarkable magnetic properties and wide-ranging applications [1]. The unique combination of high magnetic permeability, low coercivity, and excellent thermal stability makes permalloy an ideal candidate for various technological advancements, including magnetic sensors, magnetic random-access memory (MRAM), and spintronic devices [2,3]. In recent years, the exploration of permalloy at the nanoscale has gained prominence, with a particular focus on the synthesis, characterization, and manipulation of permalloy nanowires [4,5]. These nanoscale structures exhibit intriguing magnetic behaviors and offer great potential for further advancements in nanoelectronics and magnetic nanodevices.

The development of interconnected nanowire networks has garnered significant interest in the field of nanotechnology due to their potential applications in various areas. These applications include photonic devices [6], electromagnetic interference (EMI) shielding [7], thermoelectric efficiency [8,9,10,11,12,13,14], logic devices [14], magnetic field sensors [15,16], integrated neuromorphic computing [16,17,18], battery electrodes [19,20], supercapacitors [21], hydrogen generation [22], and biomedical devices [23]. Among these interconnected networks of nanowires, those formed by magnetic nanowires stand out [15,17]. Magnetic nanowires are pseudo-one-dimensional structures characterized by their typically tens to hundreds of nanometers in diameter and lengths that can reach several micrometers. The interconnection of these nanowires in a network creates complex and tunable magnetic properties. These properties can be controlled by both the geometric and the magnetic parameters of the network, as well as external stimuli [24,25,26,27].

In recent years, significant progress has been made in the synthesis and characterization of magnetic nanowire networks, as well as in the development of theoretical models to understand their properties. These advancements have paved the way for exciting possibilities in the field of nanotechnology. One approach used to achieve these nanowire networks involves the utilization of track-etched polycarbonate templates with intersecting nanochannels [9,10,11,12,13,14,15,16,17,18,19]. These templates provide a versatile platform for fabricating interconnected networks of magnetic nanowires. By carefully controlling the fabrication parameters, such as the etching time and the template thickness, it is possible to tune the dimensions and density of the nanochannels, which in turn influence the properties of the resulting nanowire network. Another alternative method involves the utilization of an aluminum oxide membrane that, when anodized, forms a three-dimensional interconnected channel structure [6,8,28,29]. This technique offers a different approach to create magnetic nanowire networks with unique characteristics. Additionally, two-photon lithography is another powerful technique capable of creating 3D polymer nanostructures. By combining this with postprocessing and deposition methods, it becomes possible to produce 3D magnetic nanostructures of any desired geometry [30]. This combination of fabrication techniques provides researchers with a wide range of options to tailor the properties of interconnected nanowire networks.

The ability to control the geometry and structure of these networks allows for the manipulation of their magnetic properties, providing opportunities for tuning their behavior in different applications. The aforementioned developments have not only expanded our understanding but also opened up new opportunities for the design and fabrication of functional devices based on these materials. These devices encompass a wide range of applications, including EMI shields [7], thermoelectric coolers [9], thermocouples [12], flexible magnetic switches for thermoelectric generation [14], and integrated multistate memristors [16,17]. However, despite the progress made, numerous challenges remain to be addressed in order to fully harness the potential of interconnected nanowire networks in practical devices.

**Figure 1:**
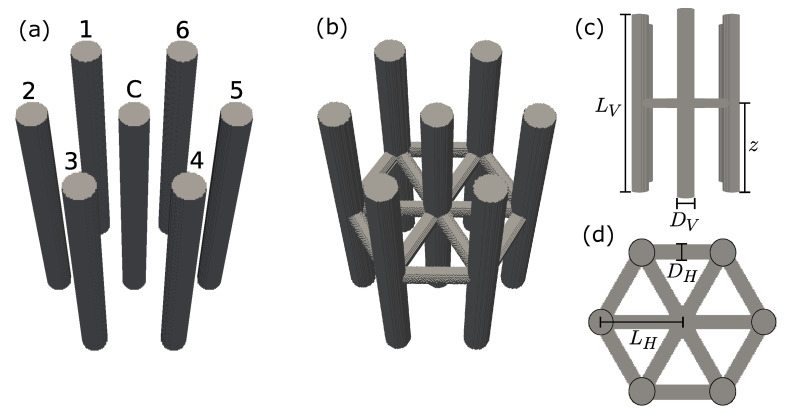
Schematic representation of the investigated nanostructures: (**a**) array of nonconnected nanowires; (**b**) array of interconnected nanowires; (**c**) side view of the array of interconnected nanowires; (**d**) top view of the array of interconnected nanowires. The numbering of the nanowires shown in (**a**) is used to identify the order in which the nanowires reverse their magnetization.

In this article, we focus on a comparative study of the magnetic properties between nonconnected arrays of permalloy nanowires and interconnected networks of permalloy nanowires. The nonconnected arrays consist of individual nanowires with dimensions of 500 nm in length and 50 nm in diameter. On the other hand, the interconnected networks are formed by horizontally oriented nanowires positioned at a specific height relative to the base surface, between the vertically aligned nanowires [31]. The horizontal diameter of the interconnected nanowires varies between 20 and 50 nm, as illustrated in Figure 1. The vertical nanowires are arranged in a hexagonal cell configuration with a center-to-center separation of 100 nm. The primary objective of this study is to investigate the dependence of the magnetic properties on the geometric parameters of the system. By systematically exploring and analyzing the effects of these parameters, we aim to gain insights into the underlying mechanisms governing the magnetic behavior of interconnected permalloy nanowire networks. This knowledge can contribute to the design and optimization of future nanoscale devices where tunable magnetic properties play a crucial role.

## 2. Micromagnetic Simulations

The magnetic properties of interconnected permalloy nanowire networks were investigated using micromagnetic simulations performed with the mumax^3^ software [32], which solves the Landau–Lifshitz–Gilbert equation (LLG) [33]. The micromagnetic simulations were performed on a computer specifically configured to work with an RTX 3090 video card, equipped with 10,752 CUDA cores, which offers exceptional computational power, making it one of the best options for conducting GPU-based micromagnetic studies. These simulations provide a powerful and reliable tool for understanding the magnetic behavior of nanoscale systems and offer valuable insights into the dynamic processes within the network.

As this study considers interconnected arrays of nanowires, we defined that our aspect ratio should not be less than 10. Considering that the pores obtained through porous alumina membranes typically have diameters of 50 nm [31], the vertically positioned nanowires have a diameter (*D_V_*) of 50 nm and a length (*L_V_*) of 500 nm, as depicted in Figure 1a. The horizontally oriented nanowires, which determine the center-to-center distance of the vertically aligned nanowires, have a length (*L_H_*) of 100 nm, which, according to Escrig et al. [34], corresponds to a moderately interacting system with *D_V_*/*L_H_* = 0.5. Additionally, the horizontally oriented nanowires exhibit two different diameters (*D_H_*): 20 nm and 50 nm. These varying dimensions allow us to explore the impact of geometry on the magnetic properties of the network.

For the micromagnetic simulations, we employed magnetic parameters specific to permalloy. The gyromagnetic ratio (γ) was set to 2.211 × 10^5^ A^−1^·s^−1^, while the saturation magnetization (M_s_) was determined to be 8 × 10^5^ A/m. The stiffness constant (A) was considered to be 13 × 10^−12^ J/m [35,36,37]. In these simulations, we did not include magnetocrystalline anisotropy for the permalloy nanowires. By neglecting this effect, we could focus on the influence of other factors, such as geometric parameters, on the magnetic behavior of the interconnected nanowire networks.

To accurately capture the nanoscale features of the system, a cubic cell size of 2 × 2 × 2 nm^3^ was utilized [38]. This cell size is sufficiently small to replicate the intricate geometry of the nanostructures and is below the exchange length of permalloy (*L_ex_* = 5.3 nm) [37]. It is important to note that this cell size, which encompasses 6,699,000 cells per simulation, necessitated running the simulations only once for each nanowire configuration. To obtain the hysteresis curves, we utilized a higher value of α = 0.5 to ensure rapid convergence, which is a commonly adopted practice in micromagnetic simulations without notable deviations observed in the results. Principio del formularioFinal del formulario The external magnetic field was applied parallel to the vertical direction of the nanowires (corresponding to the *z*-axis). A magnitude of *H* = 600 mT was used to saturate the samples, and field steps of 2 mT were used to obtain the hysteresis curves. The appropriate equilibrium configuration at every given field intensity was found by integrating the Landau–Lifshitz–Gilbert equation while the maximum torque of the system was >10^−4^ T [39,40]. By employing appropriate simulation parameters and considering the nanoscale details of the interconnected permalloy nanowire networks, we can gain valuable insights into their magnetic properties and explore the interplay between geometry and magnetism in these fascinating systems.

## 3. Results and Discussion

**Figure 2:**
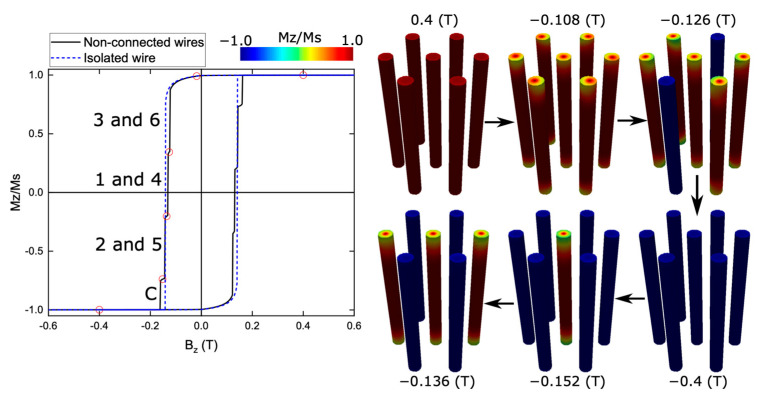
Hysteresis curve of an isolated nanowire (dashed blue line) and an array of nonconnected nanowires with a length of *L_V_* = 500 nm and a diameter of *D_V_* = 50 nm (solid black line) when the external magnetic field is applied along the *z*-axis. Snapshots of the magnetization for the highlighted red points in the hysteresis curve of an array of nonconnected nanowires are shown on the right.

In Figure 2, we present the hysteresis curve of an array of nonconnected nanowires, where each nanowire has a length of *L_V_* = 500 nm and a diameter of *D_V_* = 50 nm. The external magnetic field is applied along the *z*-axis, and the resulting hysteresis curve exhibits interesting characteristics. It displays a square shape, with a coercivity (Hc) of approximately 130 mT, representing the magnitude of the applied field needed to demagnetize the system. Furthermore, the normalized remanence (Mr/Ms) is very close to 1.0, indicating that a high proportion of magnetization is retained in the absence of an external field. Considering that permalloy is a soft magnetic material, the cause of this normalized remanence is the shape anisotropy of the nanowires, which facilitates easy magnetization along the wire axis. As a result, the magnetic moments would require a strong magnetic field in the opposite direction to induce the magnetization reversal of the nanowire.

To gain further insight into the magnetization dynamics during the hysteresis process, we provide snapshots of the magnetization for selected points along the curve. These snapshots, highlighted as red circles, are shown on the right side of the hysteresis curve. Upon close examination, we observe that the nanowires initiate the nucleation of closure domains at their tips, resulting in a slight decrease in overall magnetization within the hysteresis curve (approximately −0.108 T). As the magnetic field decreases in intensity, the magnetic moments strive to align with the wire surfaces, maintaining their direction along the wire. However, at the tips, they rotate to align with the surface of the cap, which is perpendicular to the wire axis. This leads to the formation of closure domains, which give rise to domain walls propagating from the tips toward the center of the nanowire. 

As the applied field continues to increase, we observe the occurrence of abrupt jumps in the curve, indicating the reversal of specific nanowires within the array. The first abrupt jump corresponds to the reversal of nanowires 3 and 6 (−0.126 T), followed by another jump corresponding to the reversal of nanowires 1 and 4 (−0.136 T). Subsequently, we witness the reversal of nanowires 2 and 5 (−0.152 T). These abrupt jumps in the hysteresis curve highlight the cooperative behavior and interplay between neighboring nanowires within the array. Lastly, the process of reversing the magnetization concludes with the reversal of the central nanowire (−0.4 T). By analyzing the hysteresis curve and the corresponding snapshots, we can deduce that the nanowire array exhibits three instances of double jumps and one single jump, with the central nanowire being the last to reverse its magnetization. This reversal process is expected since the nanowire configuration exhibits a sixfold symmetry. As a result, the first magnetization reversal should occur in each of the three peripheral nanowire pairs with equal probability. Similarly, the reversal order in the two remaining pairs should also be random. It is expected that, for a much larger array, the behavior should be similar to that described in Figure 1b in Escrig et al. [41], where the coercivity should remain relatively constant, but an increasing number of Barkhausen jumps associated with the reversal of groups of wires should appear, eventually leading to a continuous behavior and resulting in a decrease in remanence. 

**Figure 3:**
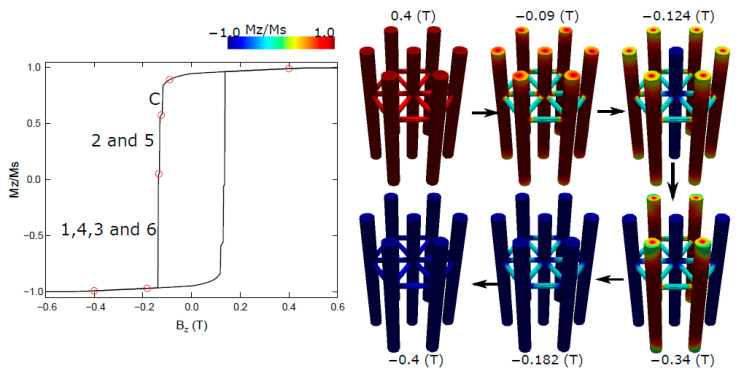
Hysteresis curve of an interconnected network of nanowires with horizontal wires of diameter *D_H_* = 20 nm located at a height of 0.65 *L_V_*. The vertical nanowires maintain a length of *L_V_* = 500 nm and a diameter of *D_V_* = 50 nm. The magnetic field is applied along the *z*-axis. Snapshots of the magnetization for the highlighted red points in the hysteresis curve are shown on the right.

In Figure 3, we present the hysteresis curve of an interconnected network of nanowires. This network consists of vertically positioned nanowires with a length of *L_V_* = 500 nm and a diameter of *D_V_* = 50 nm, while horizontally oriented nanowires with a diameter of *D_H_* = 20 nm are situated at a specific height relative to the base surface of 0.65 *L_V_* above the vertical nanowires. Similar to the previous case, the magnetic field is applied along the *z*-axis to investigate the magnetization behavior.

Upon analyzing the hysteresis curve, we observe that the overall trend of a square hysteresis curve is maintained, albeit with slight variations compared to the array of nonconnected nanowires. The interconnected network exhibits a slightly higher coercivity and a slightly lower normalized remanence. These differences arise due to the interplay between the nanowires and the presence of interwire coupling, which affects the overall magnetic behavior of the system.

To gain a deeper understanding of the magnetization dynamics within the interconnected nanowire network, we present snapshots of the magnetization for selected points along the hysteresis curve, represented by red circles. Similar to the previous case, we observe the nucleation of closure domains at the tips of the nanowires, resulting in a decrease in magnetization within the hysteresis curve (approximately −0.09 T).

However, unlike the nonconnected system, in this interconnected network, it is the central nanowire that first reverses its magnetization. This reversal of the central nanowire contributes to the observed increase in coercivity (approximately −0.124 T). Subsequently, an abrupt jump in magnetization occurs, indicating the reversal of nanowires 2 and 5 (−0.134 T). The most significant jump follows, corresponding to the reversal of nanowires 1, 3, 4, and 6 (−0.182 T). Finally, the horizontal nanowires reverse their magnetization, leading to a complete magnetization reversal of the network (−0.4 T). In summary, the hysteresis curve of the interconnected nanowire network exhibits one single jump, one double jump, and one quadruple jump, with the central nanowire being the first to reverse its magnetization.

**Figure 4:**
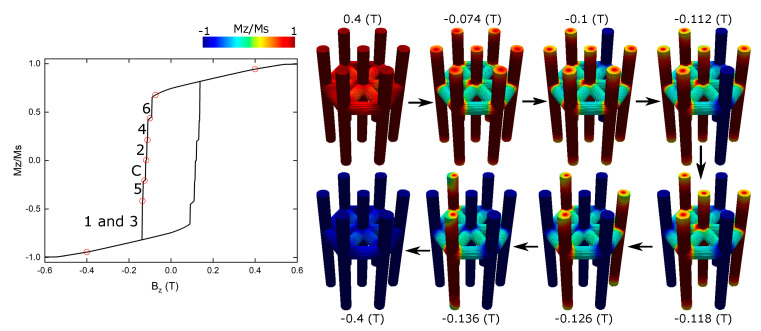
Hysteresis curve of an interconnected network of nanowires with horizontal wires of diameter *D_H_* = 50 nm located at a height of 0.65 *L_V_*. The vertical nanowires maintain a length of *L_V_* = 500 nm and a diameter of *D_V_* = 50 nm. The magnetic field is applied along the *z*-axis. Snapshots of the magnetization for the highlighted red points in the hysteresis curve are shown on the right.

In Figure 4, we present the hysteresis curve of an interconnected network of nanowires. This network comprises vertically positioned nanowires with a length of *L_V_* = 500 nm and a diameter of *D_V_* = 50 nm, while horizontally oriented nanowires with a diameter of *D_H_* = 50 nm are positioned at a specific height relative to the base surface of 0.65 *L_V_*, between the vertical nanowires. Similar to the previous cases, the magnetic field is applied along the *z*-axis to examine the magnetization behavior. Analyzing the hysteresis curve, we observe that both the coercivity and the normalized remanence decrease compared to the array of nonconnected nanowires. 

To gain further insights into the magnetization dynamics within the interconnected nanowire network, we present snapshots of the magnetization for selected points along the hysteresis curve, highlighted as red circles. Similar to the previous cases, the nanowires within the network exhibit nucleation of closure domains at their tips, leading to a reduction in the overall magnetization (−0.074 T), as depicted in the snapshots. However, in contrast to the nonconnected system, the reversal of magnetization in the interconnected network follows a different sequence.

The magnetization reversal initiates with the individual reversal of various nanowires. Nanowire 6 is the first to reverse its magnetization (−0.1 T), followed by nanowire 4 (−0.112 T) and nanowire 2 (−0.118 T). Subsequently, the central nanowire undergoes magnetization reversal (−0.126 T); finally, nanowire 5 reverses its magnetization (−0.136 T). The most significant jump in the hysteresis curve corresponds to the collective reversal of nanowires 1 and 3 (−0.4 T). Consequently, the hysteresis curve of the interconnected network exhibits a combination of five single jumps and one double jump, with the central nanowire being the fourth to reverse its magnetization.

**Figure A1:**
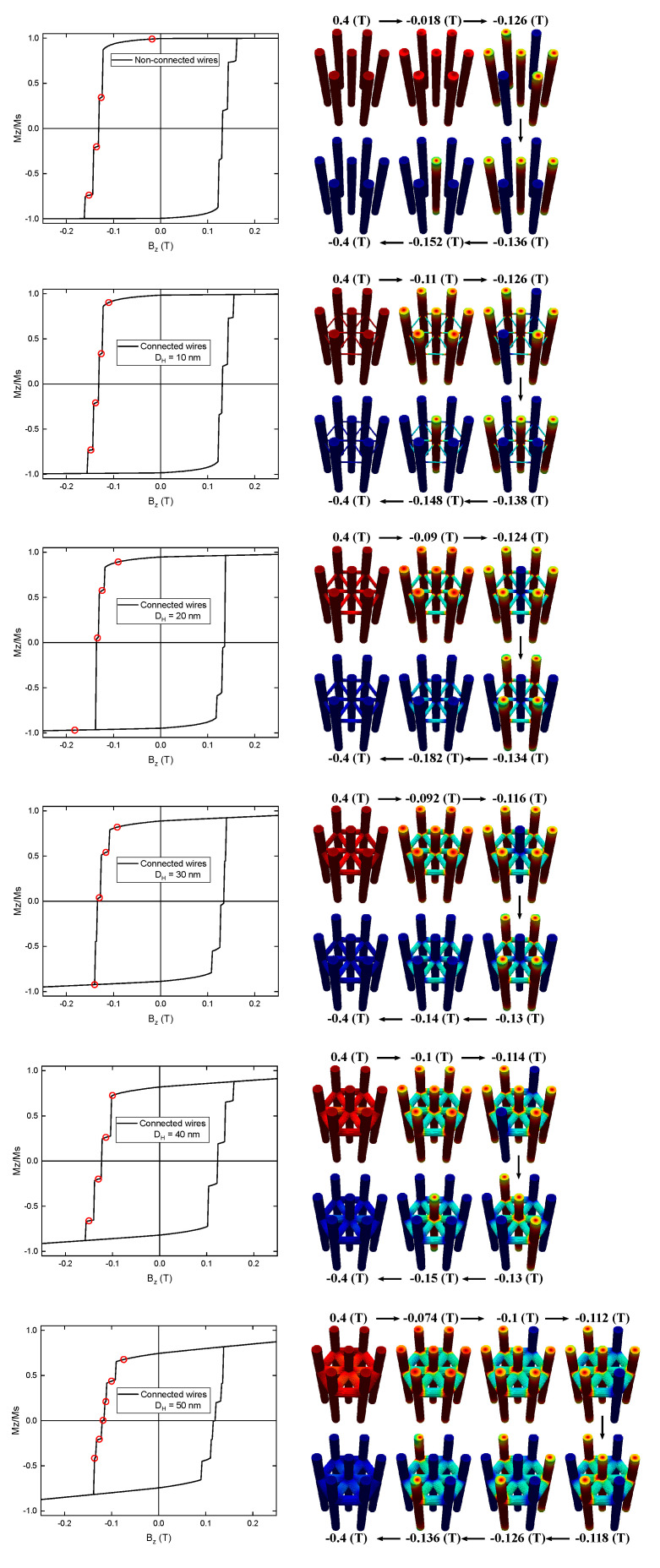
Hysteresis curves of an interconnected nanowire network with horizontal nanowires of varying diameters *D_H_* ranging from 0 to 50 nm located at a height of 0.65 *L_V_*, a length of *L_V_* = 500 nm, and a diameter of *D_V_* = 50 nm when the external magnetic field is applied along the *z*-axis. Snapshots of the magnetization for the highlighted red points in the hysteresis curve are shown on the right.

On the basis of Figure 3 and Figure 4, we can conclude that the interconnected nanowire networks break the sixfold symmetry exhibited by the nonconnected system, resulting in changes in the magnetic properties such as coercivity and remanence of the array. To delve deeper into this observation, Figure A1 in Appendix A displays the hysteresis curves of the interconnected nanowire networks as a function of the diameter of the horizontally oriented nanowires. The figure reveals that, for diameters larger than 10 nm, the symmetry of the system is indeed broken.

**Figure 5:**
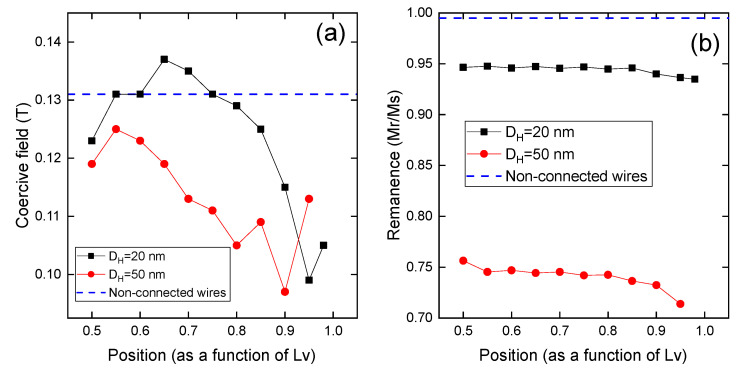
(**a**) Coercivity and (**b**) normalized remanence for arrays of nonconnected nanowires (blue dashed line), and networks of interconnected nanowires with horizontal diameter *D_H_* = 20 nm (black squares) and *D_H_* = 50 nm (red circles), as a function of the position of the horizontal nanowires relative to *L_V_*, when the external magnetic field is applied along the *z*-axis.

In Figure 5, we present the coercivity and normalized remanence characteristics for both arrays of nonconnected nanowires and networks of interconnected nanowires, as a function of the position of the horizontal nanowires relative to *L_V_*. The array of non,-connected nanowires, represented by the blue dashed line, displays a coercivity of approximately 0.13 T and a normalized remanence close to 1.0.

**Figure A2:**
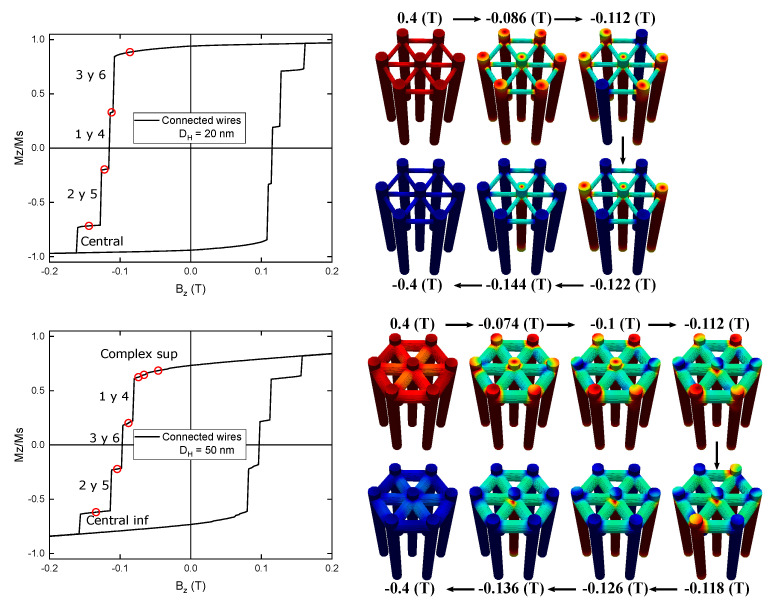
Hysteresis curves of an interconnected nanowire network with horizontal nanowires of varying diameters *D_H_* = 20 nm (top) and 50 nm (bottom), a length of *L_V_* = 500 nm, and a diameter of *D_V_* = 50 nm when the external magnetic field is applied along the *z*-axis. Snapshots of the magnetization for the highlighted red points in the hysteresis curve are shown on the right.

When incorporating horizontally oriented nanowires at different heights relative to *L_V_*, as well as their diameter *D_H_*, to form an interconnected nanowire array, the magnetic properties exhibit variations. For *D_H_* = 50 nm, illustrated by the red circles, it is noteworthy that the coercivity demonstrates a nonmonotonic behavior relative to the position of the horizontal wires. Regardless of the specific position of the horizontal nanowires relative to *L_V_*, the coercivity consistently exhibits values lower than those of the nonconnected nanowires. The lowest coercivity is obtained for *L_V_* = 0.9 (see Figure A2 in the Appendix A). This result aligns with the findings reported by Sáez et al. [36] for modulated nanowires, where they observed that the minimum in coercivity for a given value of diameter occurs when the modulation is located near the end of the modulated nanowire, but not quite at the end.

In contrast, when considering *D_H_* = 20 nm (depicted by the black squares), the overall trend aligns with the observations for *D_H_* = 50 nm. However, an intriguing finding emerges where there exists a range of positions for the horizontal nanowires relative to *L_V_*. Within this range, the system exhibits higher coercivity compared to the nonconnected array. This observation echoes the findings reported by de la Torre Medina et al. [25] for networks of interconnected nickel nanowires, further emphasizing its significance.

From an application standpoint, this finding holds considerable importance as it highlights the possibility of enhancing the coercivity of a nanowire array by strategically introducing horizontal nanowires at specific positions, such as 0.65*L_V_* and 0.7*L_V_*. Remarkably, this approach enables coercivity enhancement without necessitating alterations in the material composition of the nanowires.

Shifting our focus to the normalized remanence, we can observe that it exhibits a nearly constant value regardless of the position of the horizontal wires relative to *L_V_*. However, a notable distinction arises as the interconnected nanowires display a lower remanence in comparison to the array of non-connected nanowires, aligning with the findings reported by Ruiz-Clavijo et al. [24] for networks of interconnected cobalt nanowires. This observation can be attributed to the behavior of the bridge formed by the horizontal nanowires, which undergoes magnetization reversal concurrently with the wire caps.

Furthermore, an interesting aspect emerges as the diameter of the horizontal nanowires increases. In such cases, the remanence experiences a further decrease. This can be attributed to the larger volume of magnetic material involved in the magnetization reversal process. The increased diameter results in a greater quantity of material within the horizontal nanowires, contributing to the overall reduction in remanence.

## 4. Conclusions

In conclusion, we conducted micromagnetic simulations to investigate the magnetic properties of interconnected permalloy nanowire networks. First, we analyzed the hysteresis curve of a nonconnected nanowire array and observed a square shape with a coercivity of approximately 130 mT and a normalized remanence close to 1.0. The reversal process involved the nucleation of closure domains at the nanowire tips, followed by sequential reversals of individual nanowires until the central nanowire was reversed.

Next, we investigated the hysteresis curves of interconnected networks with horizontal nanowires of different diameters (20 nm and 50 nm). We observed that the addition of horizontal nanowires affected the coercivity and remanence. The coercivity displayed a nonmonotonic behavior as a function of the position of the horizontal nanowires relative to the vertical nanowires. In the case of a diameter of 50 nm, the coercivity was consistently lower than that of the nonconnected nanowire array. For a diameter of 20 nm, there existed a range of positions for the horizontal nanowires that resulted in higher coercivity compared to the nonconnected array.

Regarding the normalized remanence, we found that it remained relatively constant regardless of the position of the horizontal wires relative to the vertical nanowires. However, the interconnected nanowires exhibited a lower remanence compared to the nonconnected array. This decrease in remanence was attributed to the magnetization reversal of the bridge formed by the horizontal nanowires along with the wire caps. Furthermore, increasing the diameter of the horizontal nanowires further decreased the remanence due to the increased amount of magnetic material involved.

These findings have important implications for applications involving nanowire arrays, as they demonstrate the possibility of manipulating the coercivity by introducing horizontal nanowires at specific positions without altering the nanowire material composition. Additionally, the study provides insights into the influence of interconnectivity on the magnetic properties of nanowire networks and offers a valuable foundation for further exploration and optimization of these systems.

## Data Availability

Data will be made available on request.
